# Thermophilic phosphoribosyltransferases *Thermus thermophilus* HB27 in nucleotide synthesis

**DOI:** 10.3762/bjoc.14.289

**Published:** 2018-12-21

**Authors:** Ilja V Fateev, Ekaterina V Sinitsina, Aiguzel U Bikanasova, Maria A Kostromina, Elena S Tuzova, Larisa V Esipova, Tatiana I Muravyova, Alexei L Kayushin, Irina D Konstantinova, Roman S Esipov

**Affiliations:** 1 Shemyakin and Ovchinnikov Institute of Bioorganic Chemistry, Miklukho-Maklaya Str., 16/10, Moscow, GSP-7, 117997, Russia

**Keywords:** adenine phosphoribosyltransferase, catalysis, enzyme, hypoxanthine phosphoribosyltransferase, multi-enzyme cascade, nucleotides, thermophiles

## Abstract

Phosphoribosyltransferases are the tools that allow the synthesis of nucleotide analogues using multi-enzymatic cascades. The recombinant adenine phosphoribosyltransferase (*Tth*APRT) and hypoxanthine phosphoribosyltransferase (*Tth*HPRT) from *Thermus thermophilus* HB27 were expressed in *E.coli* strains and purified by chromatographic methods with yields of 10–13 mg per liter of culture. The activity dependence of *Tth*APRT and *Tth*HPRT on different factors was investigated along with the substrate specificity towards different heterocyclic bases. The kinetic parameters for *Tth*HPRT with natural substrates were determined. Two nucleotides were synthesized: 9-(β-D-ribofuranosyl)-2-chloroadenine 5'-monophosphate (2-Сl-AMP) using *Tth*APRT and 1-(β-D-ribofuranosyl)pyrazolo[3,4-*d*]pyrimidine-4-one 5'-monophosphate (Allop-MP) using *Tth*НPRT.

## Introduction

**Figure 1:**
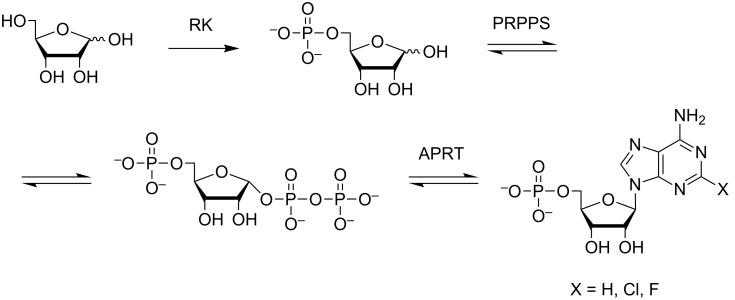
A multi-enzymatic synthesis of modified adenosine -5'-monophosphates.

Bacterial phosphoribosyltransferases are used in multi-enzymatic cascades that perform nucleotide synthesis de novo [1–2]. Recently, we reported on the possibility of cascade synthesis, where enzymes of thermophilic microorganisms *Thermus thermophilus* HB27 (phosphoribosylpyrophosphate synthetase – PRPPS and adenine phosphoribosyltransferase – APRT) and *Thermus* sp. 2.9 (ribokinase – RK) carry out successive transformations of ribose and adenine heterocyclic bases into the corresponding nucleotides (Figure 1). The use of thermophilic phosphoribosyltransferases allows carrying out reactions at a higher temperature, so the concentrations of heterocyclic bases can be increased [1–3].

There is great interest in the development of multi-enzymatic cascades [4–9] for the preparation of nucleosides and nucleotides due to the regio- and stereospecificity of enzymes [4,10–11], performing metabolic transformations of substrates. Phosphoribosyltransferases are increasingly being widely used as key enzymes in multi-enzymatic systems [2]. The substrate specificity of APRT limits the number of possible nucleotides that can be synthesized. Thus, for *Thermus thermophilus* HB27 APRT (*Tth*APRT), nucleotide synthesis is limited to the closest structural homologs of adenine (Table 1) [1].

**Table 1:** Substrates for *Tth*APRT.

Base	Conversion into nucleotide (24 h, %)^a^

2,6-diaminopurine	16.80
2-chloroadenine	97.58
2-fluoroadenine	36.50
adenine	50.02
2-methoxyadenine	60.88
N1-methyladenine	78.2
N6-benzyladenine	1.87
2-aminobenzimidazole	0.09

^a^Reaction mixtures (0.5 mL, 20 mM Tris-HCl, pH 8.0, 75 °C) contained 0.4 mM heterocyclic base, 0.4 mM PRPP, 0–5 mM MgCl_2_, 1.25 μg *Tth*APRT [1].

Unfortunately, 1,2,4-triazole-3-carboxamide, its analogues, guanine, hypoxanthine, and 7-deazapurins are not substrates for *Tth*APRT. This severely limits the usability of multi-enzymatic cascades in the synthesis of nucleotides, including the modified ones.

**Figure 2:**
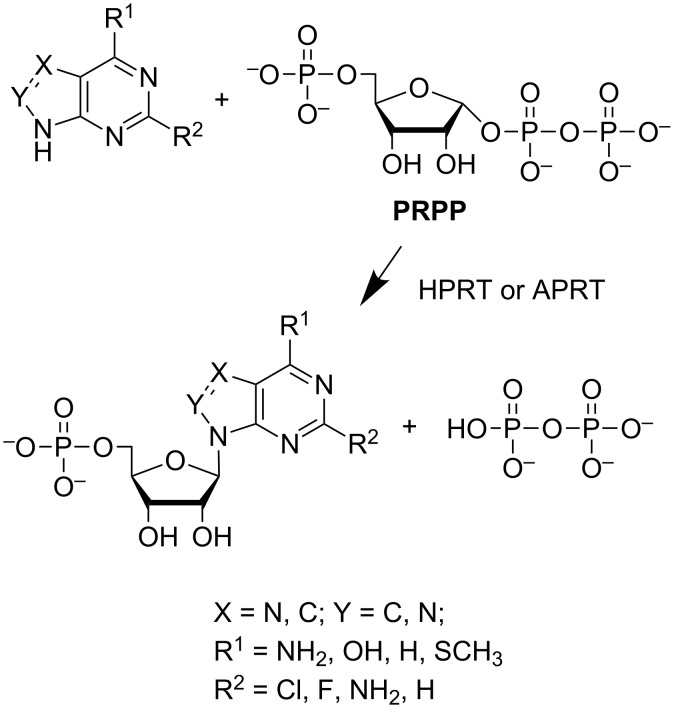
Nucleotide synthesis using phosphoribosyltransferases.

To expand the possible repertoire of nucleotides that could be synthesized, we obtained the recombinant form of hypoxathine phosphoribosyltransferase *Thermus thermophilus* (*Tth*HPRT), investigated its substrate specificity and optimal conditions for catalytic activity, and determined the kinetic parameters of the enzyme. A comparative study of the substrate specificity of *Tth*APRT and *Tth*HPRT was performed to determine the usability of thermophilic transferases in nucleotide synthesis. A scheme of purine nucleotide synthesis using *Tth*APRT and *Tth*HPRT is shown in Figure 2.

## Results and Discussion

Genes TT_RS08985 and TT_RS06315 from *T. thermophilus* HB27, coding *Tth*HPRT and *Tth*APRT, were cloned into expression plasmid vectors pET 23a+ and pET 23d+, respectively. The resulting recombinant plasmid pER-*Tth*HPRT contained fusion gene HPRT-HisTag coding *Tth*HPRT with a C-terminal His-Tag. The resulting recombinant plasmid pER-*Tth*APRT contained the gene APRT coding *Tth*APRT without any additional sequences. Nucleotide sequences of the cloned genes were verified by sequencing. The codone GGG→AGG substitution corresponding to amino-acid Arg27Gly replacement was found in the gene encoding the *Tth*HPRT.

The screening of available producer strains was performed to find strains, which produce target enzymes in soluble form. The resulting strains *E. coli* BL21(DE3)/pER- *Tth*APRT and *E. coli* C3030/pER- *Tth*HPRT produced enzymes mainly in soluble form (>80%).

The established procedure for isolation and purification of *Tth*HPRT includes heat treatment, immobilized metal affinity chromatography, final size-exclusion chromatography, and concentration. For *Tth*APRT, the protocol include heat treatment, anion exchange chromatography, hydrophobic chromatography, final size-exclusion chromatography, and concentration. Yields of both transferases were no less than 10–13 mg per liter of culture, with a purity of about 96% (as determined by SDS-PAGE).

The influence of temperature and Mg^2+^ concentration on the activity of *Tth*HPRT was investigated. The results were compared with data for adenine phosphoribosyltransferase *Thermus thermophilus*, obtained earlier [1].

**Figure 3:**
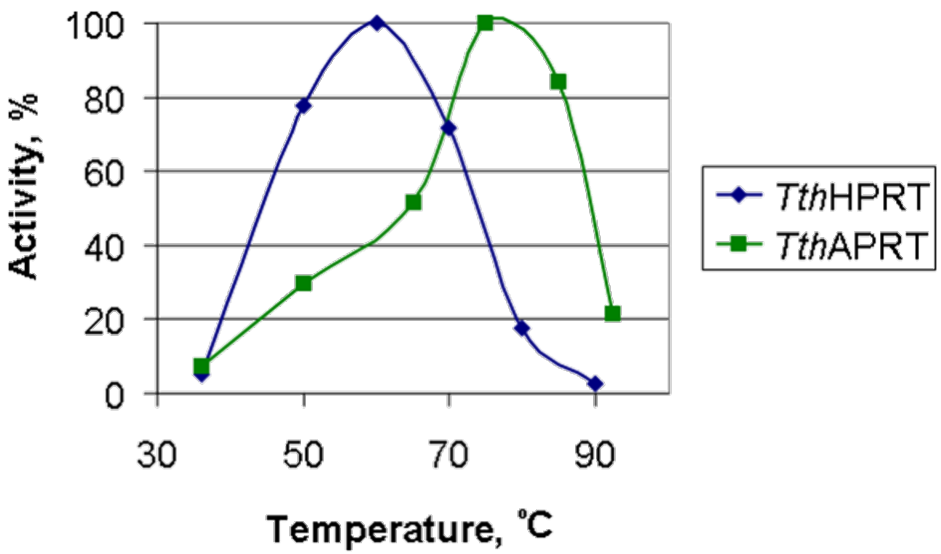
Dependence of *Tth*HPRT and *Tth*APRT activity on temperature (reaction mixtures (0.5 mL) contained 20 mM Tris-HCl, pH 8.0, 1 mM 5-phosphoribosyl-1-α-pyrophosphate, and 5 mM MgCl_2_; in the case of *Tth*HPRT mixtures contained 1 mM hypoxanthine and 0.18 μg of enzyme, in the case of *Tth*APRT – 1 mM adenine and 0.125 μg of enzyme).

The *Tth*APRT is active over a wide temperature range (Figure 3). A maximal activity of *Tth*HPRT (1.1 unit/mg) is observed at 60 °C. The activity at 36 °C is 5% from the maximal one and at 90 °C it is 3% from the maximal one. It is interesting, that *Tth*APRT shows its maximal activity at 75 °C.

**Figure 4:**
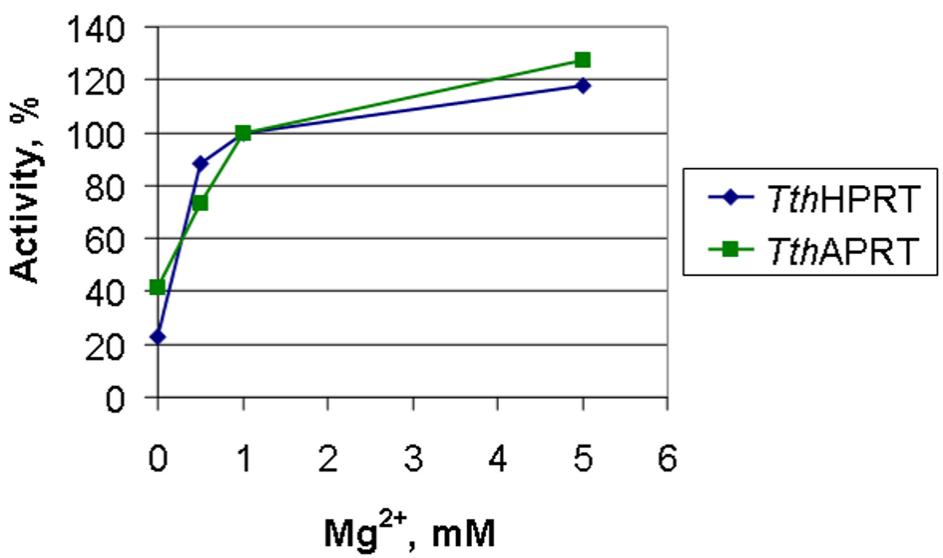
Dependence of *Tth*HPRT and *Tth*APRT activity on the Mg^2+^ concentration (reaction mixtures (0.5 mL) contained 20 mM Tris-HCl, pH 8.0, 1 mM 5-phosphoribosyl-1-α-pyrophosphate, and 0–5 mM MgCl_2_; in case of *Tth*HPRT mixtures contained 1 mM hypoxanthine and 0.18 μg of enzyme, in case of *Tth*APRT – 1 mM adenine and 0.125 μg of enzyme; reactions were performed at 70 °C; 100% – activity at 1 mM concentration).

The influence of the magnesium ion concentration on the *Tth*HPRT activity is nonlinear. The activity increases rapidly while the magnesium chloride concentration increases from 0 to 1 mM (Figure 4). Further increasing of the concentration (up to 5 mM) does not increase the activity significantly. Since the reaction rate increases rapidly with increasing the magnesium chloride concentration to values equivalent to the concentration of 5-phosphoribosyl-α-1-pyrophosphate (1 mM), it can be assumed that the presence of magnesium ions promotes the proper spatial orientation of the substrate. The reaction also proceeds in the absence of magnesium ions in solution. A similar dependence is observed for *Tth*APRT.

After optimization of the reaction conditions, kinetic parameters for *Tth*HPRT were determined (Table 2).

**Table 2:** Kinetic parameters of inosine-5'-monophosphate and guanosine-5'-monophosphate synthesis using HPRT from various organisms.

Substrate	*K*_m_, μМ	*V*_max_, μmol/min·mg	*k*_cat_, 1/s	*k*_cat_/*K*_m_, 1/M·s

*Thermus thermophilus* HB27				
hypoxanthine	13 ± 4	28 ± 9	9 ± 3	6.9 × 10^5^
guanine	28 ± 9	6 ± 2	2.0 ± 0.7	7.1 × 10^4^
PRPP	220 ± 60	17 ± 5	6 ± 2	2.7 × 10^4^

*Thermus thermophilus* HB8 [12]				
hypoxanthine	3.9 ± 1.5	–	9.1 ± 0.8	–
guanine	7.4 ± 1.7	–	18 ± 1	–
PRPP	68 ± 18	–	20 ± 2	–

*Homo sapiens* [13]				
hypoxanthine	3.8 ± 0.3	–	2.6 ± 0.6	7 × 10^5^
PRPP	19.1 ± 1.6	–	2.5 ± 0.05	1 × 10^5^

*Escherichia coli* [14]				
Hypoxanthine	37	–	–	–
PRPP	330	–	–	–

**Figure 5:**
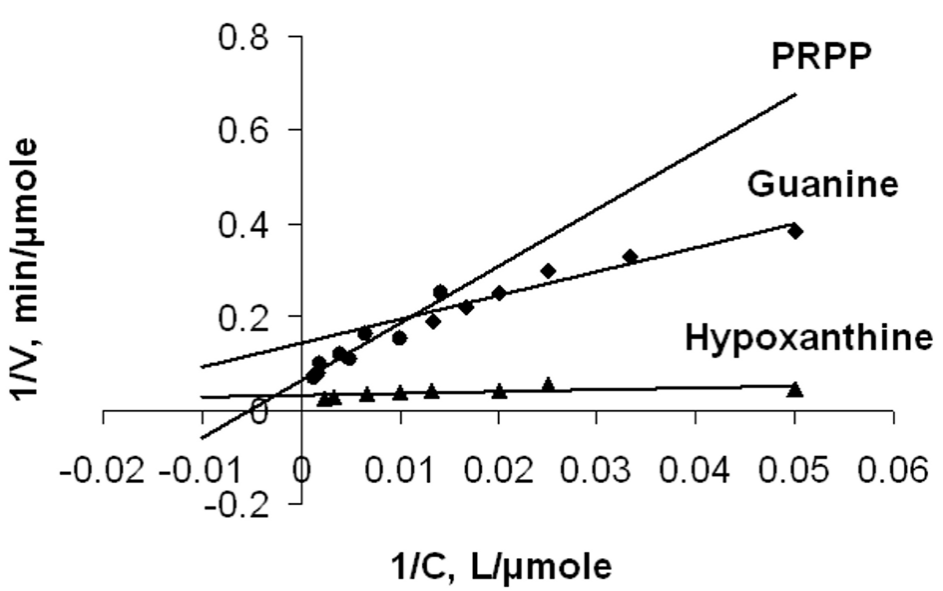
Lineweaver–Burk plot for synthesis of inosine-5'-monophosphate and guanosine-5'-monophosphate.

Based on the *K*_m_ values, the affinity of 5-phosphoribosyl-α-1-pyrophosphate for the active site is much lower than that of heterocyclic bases. The similar situation we observed for *Tth*APRT [1]. Comparison of the synthesis rates of inosine-5'-monophosphate and guanosine-5`-monophosphate showed that the first is synthesized 4.6 times faster. The literature data for similar enzymes (see Table 2) confirm a poor affinity of PRPP to the active site: *K*_m_ for hypoxanthine is 17 fold less then for PRPP, although for the human enzyme *K*_m_ is only 5 fold less. Comparing two enzymes from different strains of *Thermus thermophilus*, we can conclude that *Tth*APRT from HB8 (in contrast with HB27), synthesizes guanosine-5`-monophosphate faster. This may be due to the difference in reaction conditions. Kinetic data are displayed by double reciprocal plot (Figure 5). Determination of substrate specificity of *Tth*HPRT was performed in comparative experiments with *Tth*APRT. The process of nucleotide synthesis was monitored by a liquid chromatography–mass spectrometry analysis of the reaction mixture.

The data is presented in the Table 3. As expected, *Tth*HPRT is specific to 6-oxopurines, while *Tth*APRT is specific to 6-aminopurines. Both enzymes do not recognize thymine as a substrate. This is consistent with data that pyrimidine heterocyclic bases are substrates for uracyl phosphoribosyltransferase and orotate phosphoribosyltransferase only [2]. Unfortunately, we did not find any product in reactions with compounds based on 1,2,4-triazole-3-carboxamide, which was also observed for *E. сoli* HPRT [15–16]. However, allopurinol and 8-azaguanine are substrates for *Tth*HPRT, and 2-chloroadenine is a substrate for *Tth*APRT. For 2-chloroadenine and 8-azaguanine, reaction at a higher temperature is preferable because of their low solubility in water (less than 1 mM at 37 °C). Interestingly, allopurinol proved to be a good substrate for both *Tth*HPRT and *Tth*APRT, unlike hypoxanthine, which differs only in the position of one of the nitrogen atoms. Probably, the presence of nitrogen atom at C7 position of purine heterocycles plays an important role in reactions catalyzed by phosphoribosyltransferase, and also affects the substrate properties of *Tth*HPRT and *Tth*APRT.

**Table 3:** Substrate specifity of *Tth*HPRT and *Tth*APRT.

Base	Conversion (24 h, %)^a^	
	*Tth*HPRT	*Tth*APRT

adenine	5.3	48.1
hypoxanthine	91.0	6.4
guanine	73.9	25.6
2-chloroadenine	0	52.9
2-fluoroadenine	0	31.1
6-mercapopurine	85.1	4.8
allopurinol	39.3	57.4
8-azaguanine	80.6	1.0
thymine	0	0
1,2,4-triazole-3-carboxamide	0	0
1,2,4-triazole-3-carboxy-*N*-methylamide	0	0

^a^Reaction mixtures (0.125 mL, 20 mM Tris-HCl, pH 8.0, 60 °C) contained 0.5 mM heterocyclic base, 0.5 mM PRPP, 0.5 mM MgCl_2_, 5 mМ KH_2_PO_4_ and 0.4 μg of *Tth*HPRT or *Tth*APRT.

**Figure 6:**
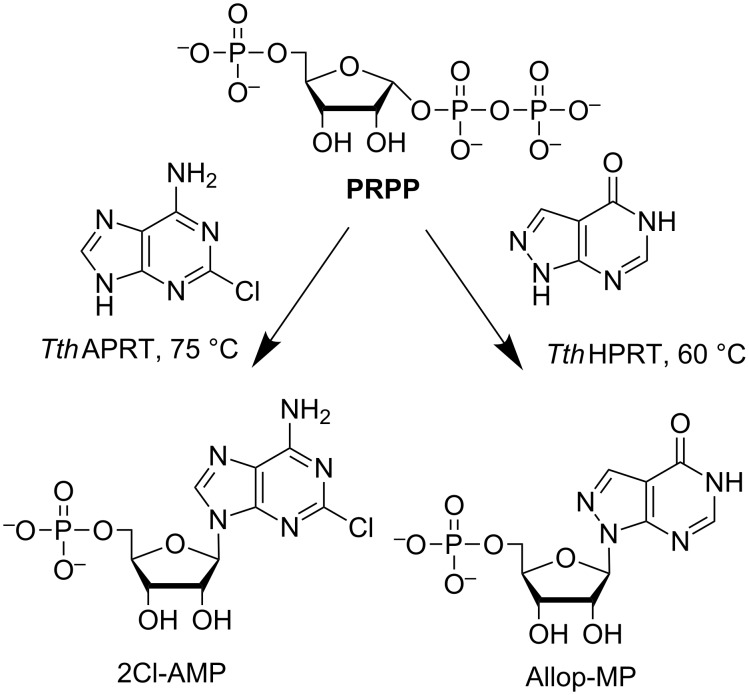
Synthesis of nucleotides 2Cl-AMP and Allop-MP using phosphoribosyltransferases *Tth*APRT or *Tth*HPRT respectively.

Two nucleotides were synthesized using *Tth*HPRT or *Tth*APRT (see Figure 6). Synthesis of 2-Cl-AMP was performed at 75 °C. This allowed to achieve a concentration of 0.5 mM of the initial 2-chloroadenine. The reaction progress was monitored by HPLC. After 2 days (the product content in the reaction mixture was 54%), the reaction mixture was concentrated and the desired product was isolated by column chromatography on ion-exchange sorbents (anion and then cation-exchange). The yield of 2-Сl-AMP was 37%. A second nucleotide (Allop-MP) was synthesized at a lower temperature (60 °C). After 2 days, the product content in the reaction mixture was 55%. The product was isolated in the same way, with a yield of 32%.

## Conclusion

The recombinant adenine phosphoribosyltransferase and hypoxanthine phosphoribosyltransferase from *Thermus thermophilus* HB27 were purificated with yields no less than 10–13 mg per litre of culture. A comparative study of substrate specificity of these enzymes towards different heterocyclic bases was carried out and temperature-dependence and magnesium chloride concentration-dependence of enzymes activity were determined. *Tth*HPRT can be used for the synthesis of nucleotides containing different purine derivatives including 8-aza- and 8-aza-7-deazapurine. The use of hypoxanthine and adenine transferases in multi-enzyme cascades significantly extends the spectrum of synthetic purine nucleotides. Two nucleotides were synthesized: 9-(β-D-ribofuranosyl)-2-chloroadenine 5'-monophosphate (2-Сl-AMP) using *Tth*APRT and 1-(β-D-ribofuranosyl)pyrazolo[3,4-*d*]pyrimidine-4-one 5'-monophosphate (Allop-MP) using *Tth*НPRT with yields of 37% and 32%, respectively. Using of hypoxanthine and adenine transferases in multi-enzyme cascades significantly extends the spectrum of synthetic purine nucleotides.

## Experimental

Tris-buffer, acetic acid, sodium chloride, glycerol, acrylamide, *N*,*N*'-bisacrylamide, ATP, bromophenol blue, agarose, EDTA, IPTG, ampicillin, sodium dodecylsulfate, imidazole and DMF were purchased from Panreac (Spain, Barselona). Ethanol was purchased from MedChemProm. Coomassie Brilliant Blue R-250 was purchased from Bio-Rad (USA, CA). Bacto yeast extract, bacto tryptone, and bacto agar were purchased from Becton Dickinson Biosciences (USA, NJ). NaOH and HCl were purchased from Merck (USA, MA). Sodium persulfate, TEMED, ethidium bromide, and sodium azide were purchased from Helicon (Russia). dNTP was purchased from Thermo Fisher Scientific (USA, MA). DTT, phenylmethylsulfonyl chloride, magnesium chloride, nickel sulfate, potassium dihydroorthophosphate, Ni-IDA sepharose, 5-phosphoribosyl-α-1-pyrophosphate and all bases (adenine, hypoxanthine, guanine, 2-chloroadenine, etc.) were purchased from Sigma-Aldrich (USA, MO).

Bacterial strains: a) *E. coli* C3030 [MiniF *lysY* (Cam^R^) / *fhuA2 lacZ::T7 gene1* [lon] *ompT ahpC gal λatt*::pNEB3-r1-*cDsbC* (Spec^R^, lacI^q^) *ΔtrxB sulA11 R(mcr-73::miniTn1*0--Tet^S^)2 [dcm] *R(zgb-210::Tn10* --Tet^S^) *endA1 Δgor ∆(mcrC-mrr)114::IS10*] New England Biolabs (USA, MA), b) *E.coli* BL21(DE3) *fhuA2 [lon] ompT gal (λ DE3) [dcm] ΔhsdS λ DE3 = λ sBamHIo ΔEcoRI-B int::(lacI::PlacUV5::T7 gene1) i21 Δnin5.*

Plasmid vector: pET 23a+, pET 23d+ (Merck Millipore, USA, MA).

Enzymes: NdeI, XhoI, NcoI, T4 DNA-ligase (Thermo Scientific, USA, MA), Encyclo-polymerase (Eurogen, Russia).

The protein concentration was determined by the Bradford method [17], using BSA as a standard.

Protein purity was determined by electrophoresis in a polyacrylamide gel under denaturing conditions [18].

**Cloning and creation of producer strain:** Genes TT_RS08985 and TT_RS06315, encoding *Tth*HPRT and *Tth*APRT, respectively, were amplified on the genomic DNA template of the *T. thermophilus* HB27 strain by a polymerase chain reaction (PCR) using synthetic primers. The genes were cloned into the expression vectors pET-23a+ and pET-23d+ respectively. The *E. coli* strains BL21(DE3)/pER- *Tth*APRT and C3030/pER- *Tth*HPRT produced the target enzymes mainly in soluble form (culturing conditions: 4 h grow at 37 °С after supplementing with 0.4 mM IPTG).

**Isolation and purification of *Tth*HPRT:** A cell pellet was resuspended in 50 mM Tris-HCl, 200 mМ NaCl, and 1 mМ phenylmethylsulfonyl fluoride (PMSF) рН 8.0 (1:10 w/v). The cells were disrupted by sonication for 30 min at 20 kHz at +4 °С. The cell debris was pelleted by centrifugation at 12,000 rpm for 30 min at +4 °С. The cell lysate was heat-treated (65 °С, 10 min) and the pellet was removed by centrifugation. The supernatant was applied to a column XK 16/20 (GE Healthcare, USA) packed with Ni-IDA Sepharose (Sigma Aldrich, USA) pre-equilibrated with 50 mM Tris-HCl and 200 mM NaCl at pH 8.0. Ballast proteins were eluted with a solution, containing 50 mM Tris-HCl, 50 mM imidazole, and 200 mM NaCl, pH 8.0 (4 CV, flow rate 2 mL/min). The target enzyme was eluted with solution, contained 50 mM Tris-HCl, 250 mM imidazole, and 200 mM NaCl, pH 8.0 (4 CV, flow rate 2 mL/min). Pooled fractions were concentrated by a polysulfonic membrane PBGC 10 kDa (Millipore, USA). The resulting solution was applied to a column with HiLoad 16/60 Superdex 75pg (GE Healthcare, USA), equilibrated by 20 mM Tris-HCl, 50 mM NaCl, 0.04% NaN_3_, and 10% glycerol, pH 8.0. Fractions, containing the target enzyme with a purity of more than 96 %, were pooled and concentrated up to a concentration of 13 ± 1 mg/mL.

**Isolation and purification of *Tth*APRT**: Cell biomass disruption and heat-treatment was performed as described in section "Isolation and purification of *Tth*HPRT". The resulting solution was diluted (2-fold) with solution, comtained 50 mM Tris-HCl, 2 M (NH_4_)_2_SO_4_, pH 8.0, and applied to column XK 16/20 packed with Phenyl Sepharose HP (GE Healthcare, USA). The column was eluted by linear gradient of (NH_4_)_2_SO_4_ (1.0 – 0 M, 12 CV, flow rate 2 mL/min). Fractions, contained the target enzyme, were pooled and concentrated on polysulphonic membrane PBGC 10 kDa. The resulting solution was applied to column with HiLoad 16/60 Superdex 200, equilibrated by 20 mM Tris-HCl, 50 mM NaCl, 0.04% NaN_3_, and 5% glycerol, pH 8.0. Fractions, contained the target enzyme with purity more than 96%, were pooled and concentrated up to concentration 12 ± 1 mg/mL.

**Enzyme assay:** Each reaction mixture (0.5 mL, 20 mM Tris-HCl, pH 8.0) contained 1 mM 5-phosphoribosyl-1-α-pyrophosphate, 1 mM hypoxanthine, 5 mM MgCl_2_, and hypoxanthine phosphoribosyltransferase *Thermus thermophilus* (0.18 μg). Reaction mixtures were incubated at 70 °C. Substrate and product quantities were determined using HPLC (Waters 1525, column Ascentis Express C18, 2.7 μm, 3.0 × 75 mm, eluent A 0.1% aqeous TFA, eluent B 0.1% TFA / 70% acetonitrile in water, detection at 254 nm, Waters 2489).

**Kinetic parameters determination:** Each reaction mixture (1.0 mL, 20 mM Tris-HCl, pH 8.0) contained 5 mM MgCl_2_, hypoxanthine phosphoribosyltransferase *Thermus thermophilus* (0.18 μg), and the following components: a) hypoxanthine (0.01–0.50 mM) or guanine (0.01–0.20 mM) and 1 mM 5-phosphoribosyl-1-α-pyrophosphate to determine *K*_m_ and *V*_max_ for hypoxanthine and guanine, and b) 5-phosphoribosyl-1-α-pyrophosphate (0.05–1.20 mM) and 0.50 mM hypoxanthine to determine *K*_m_ and *V*_max_ for 5-phosphoribosyl-1-α-pyrophosphate. Reaction mixtures were incubated at 70 °C for 2 min. Product quantities were determined as described in the "Enzyme assay" section. Each experiment was repeated three times. Kinetic parameters were determined by nonlinear regression analysis using SciDAVis v0.2.4 software (free software, web site: scidavis.sourceforge.net). Catalytic constants (*k*_cat_) were calculated per 1 subunit (20.3 kDa, calculated based on amino acid sequence).

Mass spectra were measured on an Agilent 6224, ESI-TOF, LC/MS (USA) in positive ion mode (ESI), LCQ Fleet ion trap mass spectrometer (Thermo Electron, USA) and Agilent 1100 LC/MSD VL (Agilent Technologies*)* equipped an APCI and ESI source (positive and negative mode of ionization), 1100 DAD and ELSD PL-ELS 1000 (Polymer Laboratories).

### Nucleotides synthesis

**9-(β-D-Ribofuranosyl)-2-chloroadenine 5'-monophosphate (2-Сl-AMP):** 2-Chloroadenine (17 mg, 0.10 mmol) was dissolved in water (203 mL) under stirring and heating at 90 °C , and after cooling to 70 °C, magnesium chloride hexahydrate (41 mg, 0.21 mmol) and potassium dihydroorthophosphate (276 mg, 2.03 mmol) were added. The pH of the solution was adjusted to 8.0 by 2 N potassium hydroxide. The pentasodium salt of 5-phosphoribosyl-α-1-pyrophosphate (70 mg, 0.14 mmol) and *Tth*APRT (5 units) were added, and the reaction mixture was incubated at 75 °C for 2 days; the reaction progress was monitored by HPLC. The reaction mixture was neutralized with 2 N hydrochloric acid and concentrated in vacuo to ca. 10 mL. The precipitate was filtered off, the filtrate was applied to the column with DEAE-Toyopearl 650C, bicarbonate form, 40 × 140 mm, and the product was eluted with triethylammonium bicarbonate (0.1 M). Fractions were concentrated in vacuo to ca. 10 mL, applied to the column with CM-Sephadex C-25, sodium form, 20 × 160 mm, and the product was eluted with water to give, after evaporation and drying in vacuo under P_2_O_5_, 16 mg (0.037 mmol; 37%) of 9-(β-D-ribofuranosyl)-2-chloroadenine 5'-monophosphate of 99% purity (HPLC). HRMS (ESI^+^): *m/z* [M + H]^+^ calcd for C_10_H_13_N_5_O_7_P_1_Cl_1_: 382.0315; found, 382.0353; [2M + H]^+^, found, 763.0606; [Base + H]^+^, found, 170.0244; ^1^Н NMR (DMSО-*d*_6_) δ ppm) 8.52 (s, 1Н, H8), 7.78 (br. s., 2H, NH_2_), 5.83 (d, *J*_1’,2’_ = 6 Hz, 1Н, H1’), 4.61 (m, 1Н, H2’), 4.23 (m, 1Н, H3’), 4.06 (m, 1Н, H4’), 3.84 (m, 2Н, H5a’, H5b’) ppm; ^13^C NMR (DMSО-*d*_6_) δ 156.61 (C2 or C6), 153.01 (C6 or C2), 150.73 (C4), 139.68 (C8), 117.57 (C5), 86.40 (C1’), 84.52 (C4’), 74.13 (C2’), 71.06 (C3’), 3.94 (C5’) ppm; ^15^N NMR (DMSО-*d*_6_) δ 242.7 (N7), 171.3 (N9), 86.84 (NH_2_) ppm.

**1-(β-D-Ribofuranosyl)pyrazolo[3,4-*d*]pyrimidine-4-one 5'-monophosphate (Allop-MP):** Allopurinol (14 mg, 0.10 mmol) was dissolved in water (203 mL) under stirring and heating at 90 °C, and after cooling to 50 °C, magnesium chloride hexahydrate (41 mg, 0.21 mmol) and potassium dihydroorthophosphate (276 mg, 2.03 mmol) were added. The pH of the solution was adjusted to 8.0 with 2 N potassium hydroxide. The pentasodium salt of 5-phosphoribosyl-α-1-pyrophosphate (70 mg, 0.14 mmol) and *Tth*HPRT (5 units) were added, and the reaction mixture was incubated at 60 °C for 2 days; the reaction progress was monitored by HPLC. The reaction mixture was neutralized by 2 N hydrochloric acid and concentrated in vacuo to ca. 10 mL. The precipitate was filtered off, the filtrate was placed on the column with DEAE-Toyopearl 650C, bicarbonate form, 40 × 140 mm, and the product was eluted with triethylammonium bicarbonate (0.2 M). Fractions were concentrated in vacuo to ca. 10 mL, applied to the column with CM-Sephadex C-25, sodium form, 20 × 160 mm, and the product was eluted with water to give, after evaporation and drying in vacuo under P_2_O_5_, 11 mg (0.032 mmol; 32%) of 1-(β-D-ribofuranosyl)pyrazolo[3,4-*d*]pyrimidine-4-one 5'-monophosphate of 97% purity (HPLC). HRMS (ESI^+^): *m/z* [M + H]^+^ calcd for C_10_H_13_N_4_O_8_P_1_: 349.0545; found, 349.0520; [2M + H]^+^, found, 697.0952; [3M + H]^+^, found, 1045.1374; [Base + H]^+^ found, 137.0453; ^1^Н NMR (DMSО-*d*_6_) δ 12.44 (br. s, 1H, NH), 8.15 (s, 1H, H3), 8.13 (s, 1H, H6), 6.06 (d, *J* = 4.1 Hz, 1H, H1’), 4.56 (dt, 1H, H2’, *J* = 4.54; <0.5), 4.31 (t, 1H, H3’, *J* = 4.8), 4.04 (m, 1H, H4’), 3.85 (ddd, *J* = 11.0, 7.6; 6.2 Hz, 1H, H5’a), 3.66 (ddd, *J* = 11.0, 7.2, 6.1 Hz 1H, H5’b) ppm; ^13^C NMR (DMSО-*d*_6_) δ 157.03 (C4), 152.90 (C7a), 148.53 (C6), 135.38 (C3), 106.06 (C4a), 88.16 (C1’), 83.27 (C4’), 73.39 (C2’), 71.38 (C3’), 64.76 (C5’) ppm; ^15^N NMR (DMSО-*d*_6_) δ 302.8 (N2), 210.6 (N7), 204.9 (N1), 171.1 (N5).

## Abbreviations

APRT – adenine phosphoribosyltransferase; HPRT – hypoxathine phosphoribosyltransferase; PRPPS – phosphoribosylpyrophosphate synthetase; RK – ribokinase; *Tth* - *Thermus thermophilus;* 2-Сl-AMP – 9-(β-D-ribofuranosyl)-2-chloroadenine 5'-monophosphate; Allop-MP – 1-(β-D-ribofuranosyl)-pyrazolo[3,4-*d*]pyrimidine-4-one 5'-monophosphate

## Supporting Information

File 1 Detailed analysis of mass spectrometry and NMR data.
