# Supplementary material for: Thermophilic phosphoribosyltransferases Thermus thermophilus HB27 in nucleotide synthesis
Source: Beilstein J Org Chem. 2018 Dec 21;14:3098–105. doi: 10.3762/bjoc.14.289 (PMC6317416; doi:10.3762/bjoc.14.289)
Supplement: File 1 — Detailed analysis of mass spectrometry and NMR data. [file Beilstein_J_Org_Chem-14-3098-s001.pdf]

BEILSTEIN JOURNAL OF ORGANIC CHEMISTRY

Supporting Information

for

Thermophilic phosphoribosyltransferases *Thermus thermophilus* HB27 in nucleotide synthesis

Ilja V. Fateev, Ekaterina V. Sinitsina, Aiguzel U. Bikanasova, Maria A. Kostromina, Elena S. Tuzova, Larisa V. Esipova, Tatiana I. Muravyova, Alexei L. Kayushin, Irina D. Konstantinova and Roman S. Esipov

Beilstein J. Org. Chem. **2018,** *14,* 3098–3105. doi:10.3762/bjoc.14.289

Detailed analysis of mass spectrometry and NMR data

License and Terms: This is a supporting information file under the terms of the Creative Commons Attribution License (http://creativecommons.org/licenses/by/4.0). Please note that the reuse, redistribution and reproduction in particular requires that the authors and source are credited.
The license is subject to the *Beilstein Journal of Organic Chemistry* terms and conditions: (https://www.beilstein-journals.org/bjoc)

Table of Contents

1 – Mass spectra of nucleotides (Agilent 6224, ESI-TOF, LC/MS) S2

2 – Mass spectra of nucleotides (Agilent 1100 LC/MSD VL) S4

3 – NMR data of 2-Cl-AMP S5

4 – NMR data of Allop-MP S7

5 – HPLC examples for nucleosides formation S9

6 – Nucleotide sequence and corresponding amino acid sequence S11

7. – SDS-PAGE examples S12

1. Mass spectra of nucleotides were measured on Agilent 6224, ESI-TOF, LC/MS (USA) in positive ion mode (ESI), LCQ Fleet ion trap mass spectrometer (Thermo Electron, USA).

1.1. 9-(β-*D*-ribofuranosyl)-2-chloroadenine 5'-monophosphate
$C_{10}H_{13}N_5O_7P_1Cl_1$
Calc. $[M+H]^+$ = 382.0315
Exp. $[M+H]^+$ = 382.0353, $[2M+H]^+$ = 763.0606, $[Base+H]^+$ = 170.0244

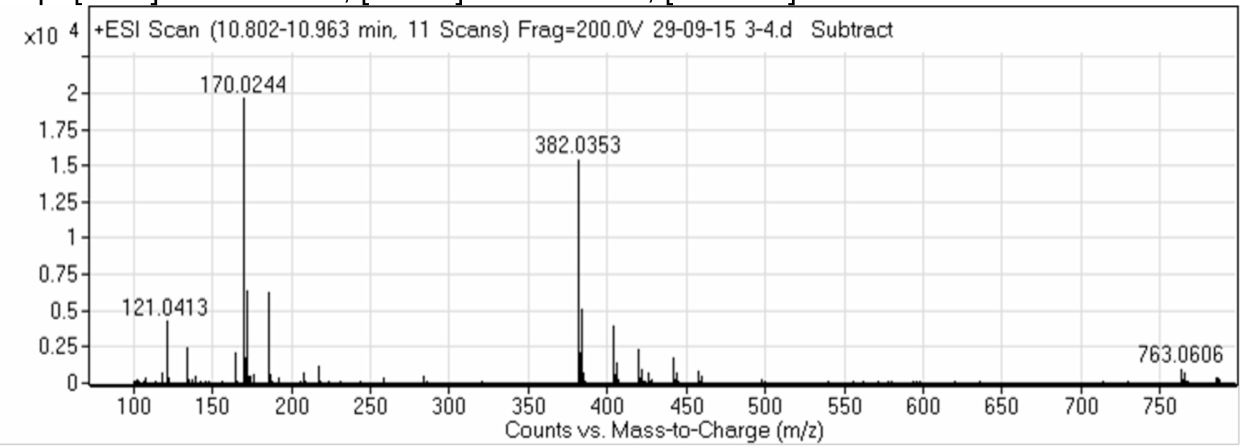

Figure S1

1.2. 9-(β-*D*-ribofuranosyl)-2-fluoroadenine 5'-monophosphate
$C_{10}H_{13}N_5O_7P_1F_1$
Calc. $[M+H]^+$ = 366.0611
Exp. $[M+H]^+$ = 366.0564, $[2M+H]^+$ = 731.1034, $[Base+H]^+$ = 154.0511

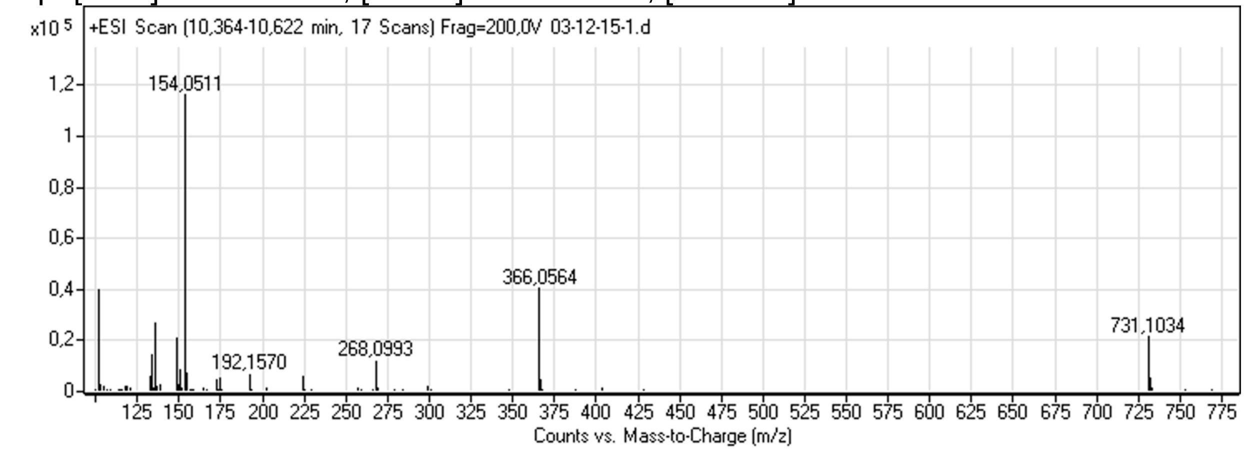

Figure S2

1.3. 1-(β-*D*-ribofuranosyl)-pyrazolo[3,4-d]pyrimidine-4-one 5'-monophosphate
$C_{10}H_{13}N_4O_8P_1$
Calc. $[M+H]^+$ = 349.0545
Exp. $[M+H]^+$ = 349.0520, $[2M+H]^+$ = 697.0952, $[3M+H]^+$ = 1045.1374, $[Base+H]^+$ = 137.0453

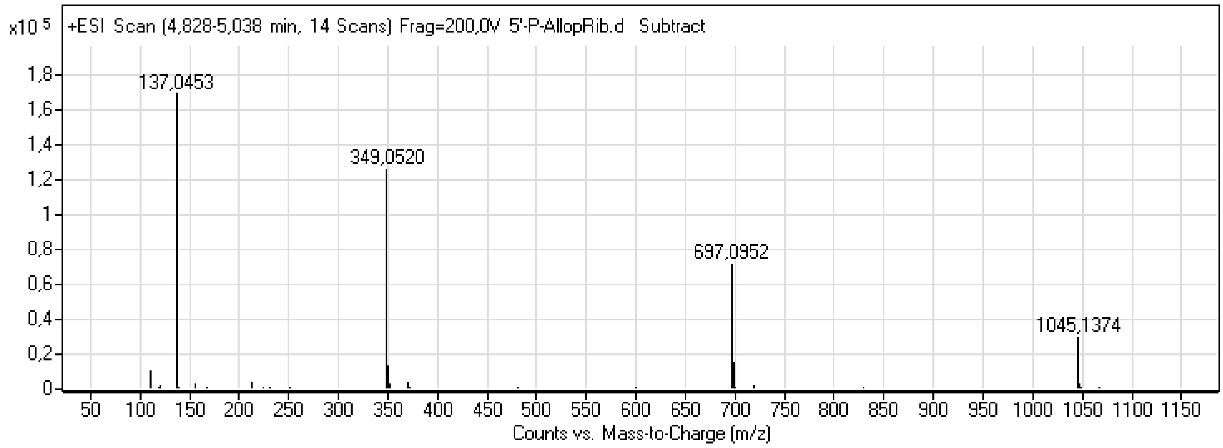

Figure S3

1.4. Adenosine 5'-monophosphate

$C_{10}H_{14}N_5O_7P_1$

Calc. $[M+H]^+$ = 348.0705

Exp. $[M+H]^+$ = 348.0677, $[2M+H]^+$ = 695.1258, $[Base+H]^+$ = 136.0607

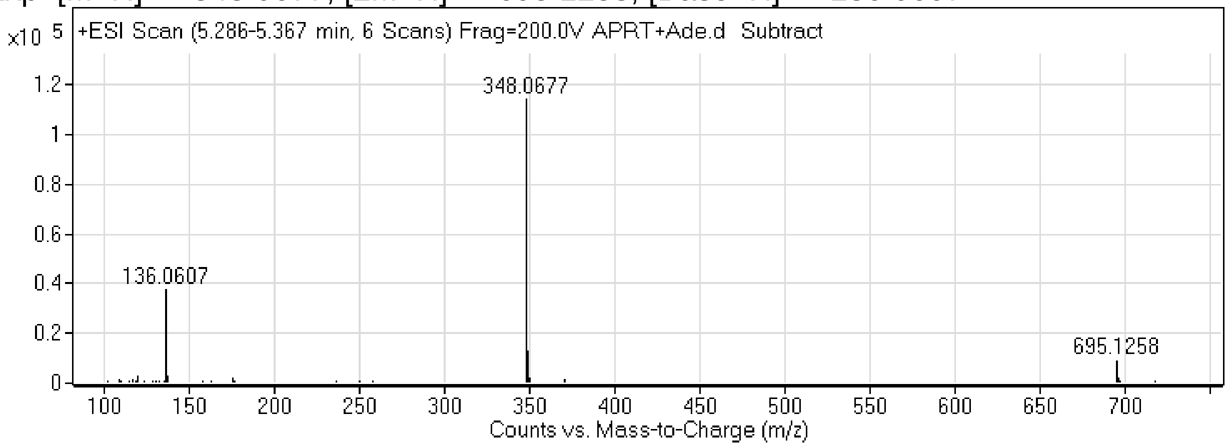

Figure S4

1.5. Guanosine 5'-monophosphate

$C_{10}H_{14}N_5O_8P_1$

Calc. $[M+H]^+$ = 364.0654

Exp. $[M+H]^+$ = 364.0614, $[2M+H]^+$ = 727.1128, $[Base+H]^+$ = 152.0557

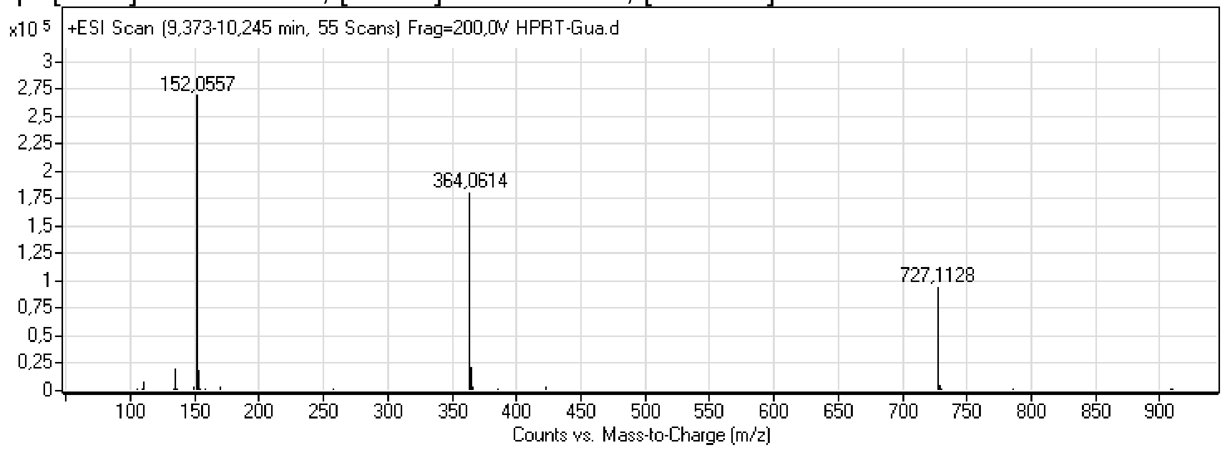

Figure S5

2. Mass spectra were measured on Agilent 1100 LC/MSD VL (Agilent Technologies) equipped APCI and ESI source in negative mode of ionization, 1100 DAD and ELSD PL-ELS 1000 (Polymer Laboratories).

2.1. 9-(β-D-ribofuranosyl)-6-mercaptopurine 5'-monophosphate
$C_{10}H_{13}N_4O_7P_1S_1$
Calc. $[M]^-$ = 363.0
Exp. $[M]^-$ = 362.8

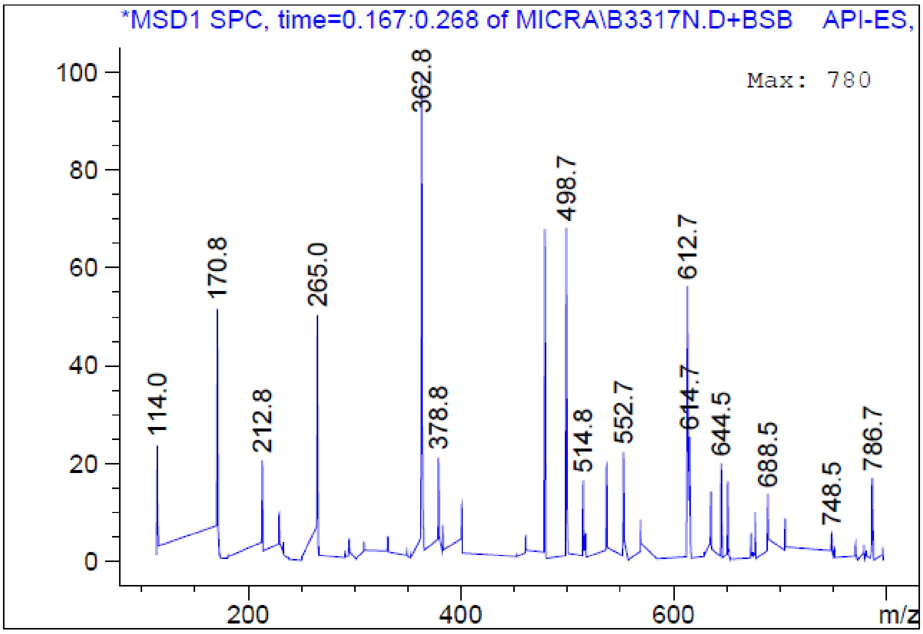

Figure S6

2.2. 5-Amino-3-(β-D-ribofuranosyl)-1,2,3-triazolo[4,5-d]pyrimidin-7-one 5'-monophosphate
$C_9H_{13}N_6O_8P_1$
Calc. $[M]^-$ = 363.0
Exp. $[M]^-$ = 362.8

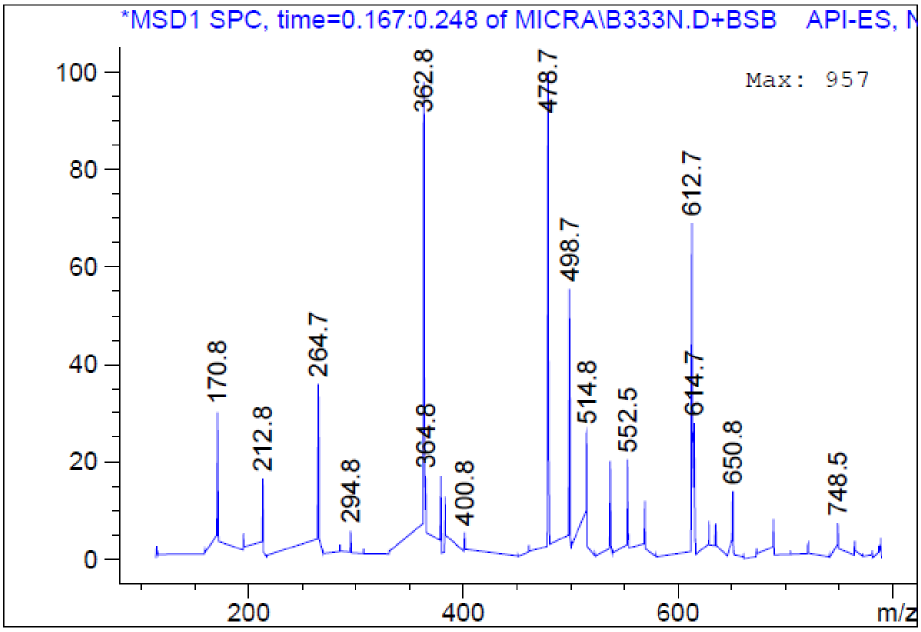

Figure S7

2.3. Inosine 5'-monophosphate

$C_{10}H_{13}N_4O_8P_1$

Calc. $[M]^-$ = 347.0

Exp. $[M]^-$ = 347.0

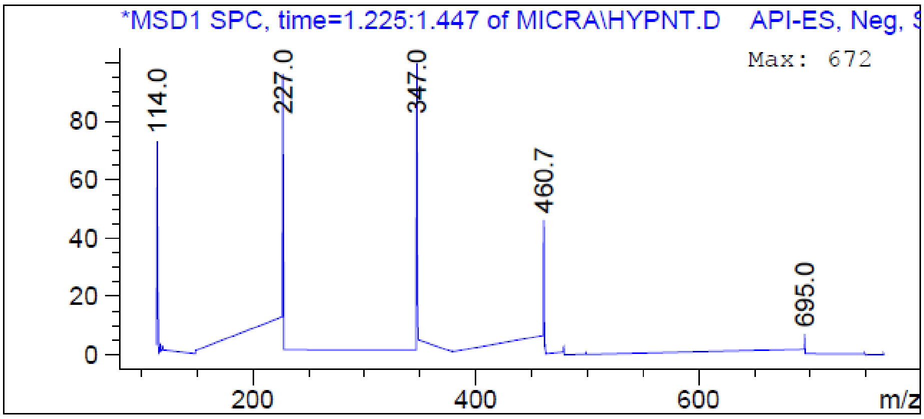

Figure S8

3. NMR data of 2-Cl-AMP

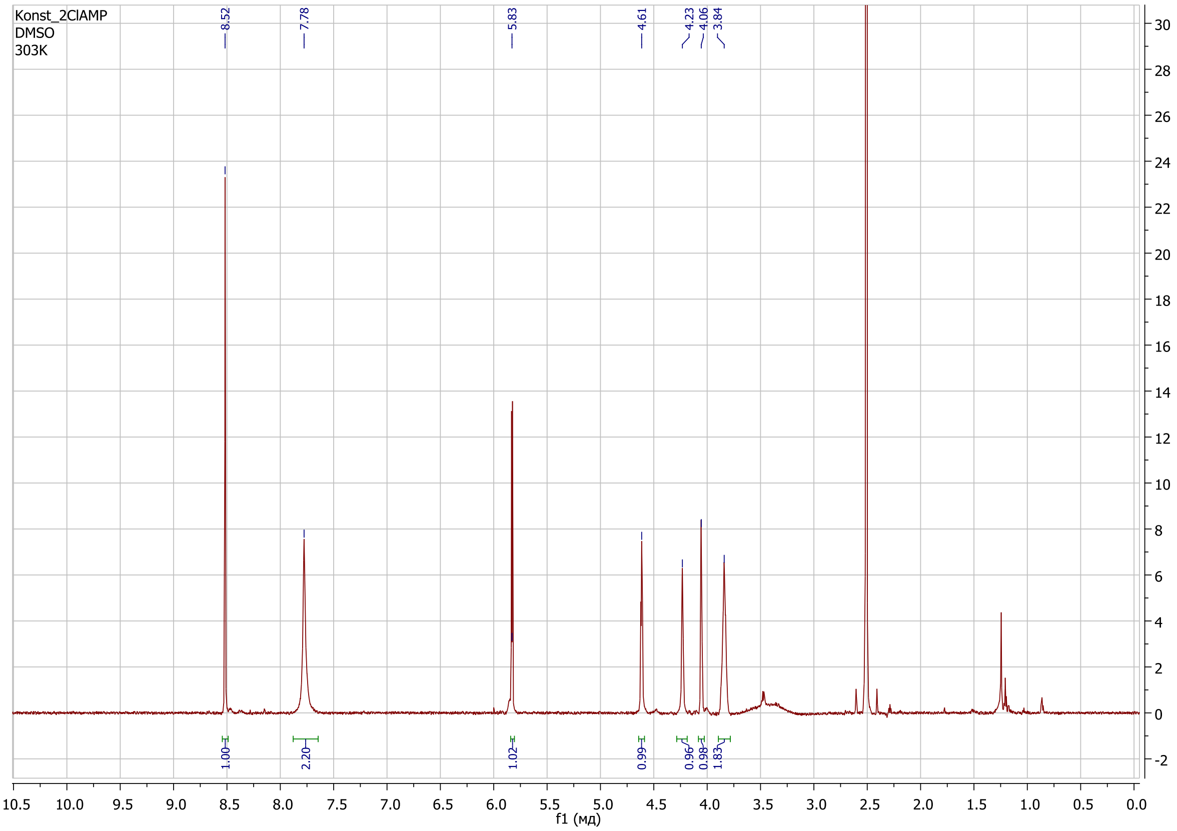

Figure S9. ^{1}H NMR spectrum of 2-Cl-AMP

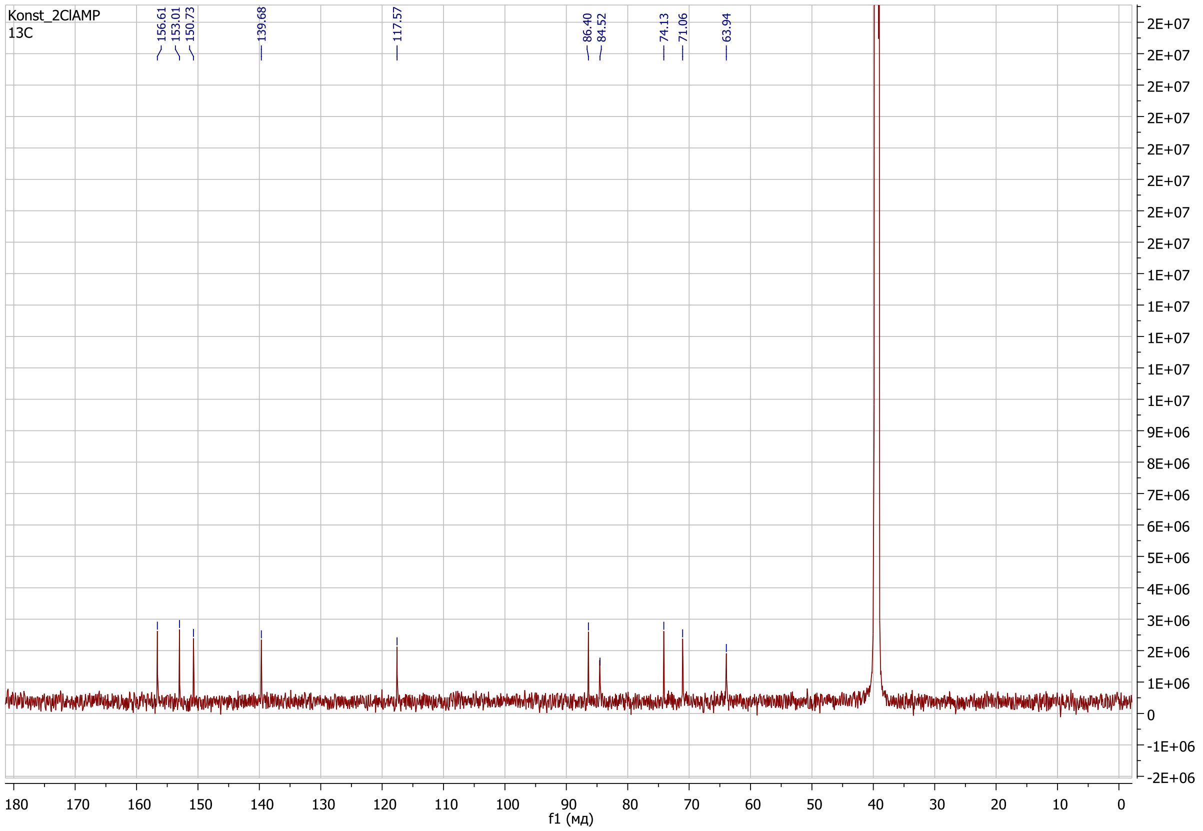

Figure S10. ^{13}C NMR spectrum of 2-Cl-AMP

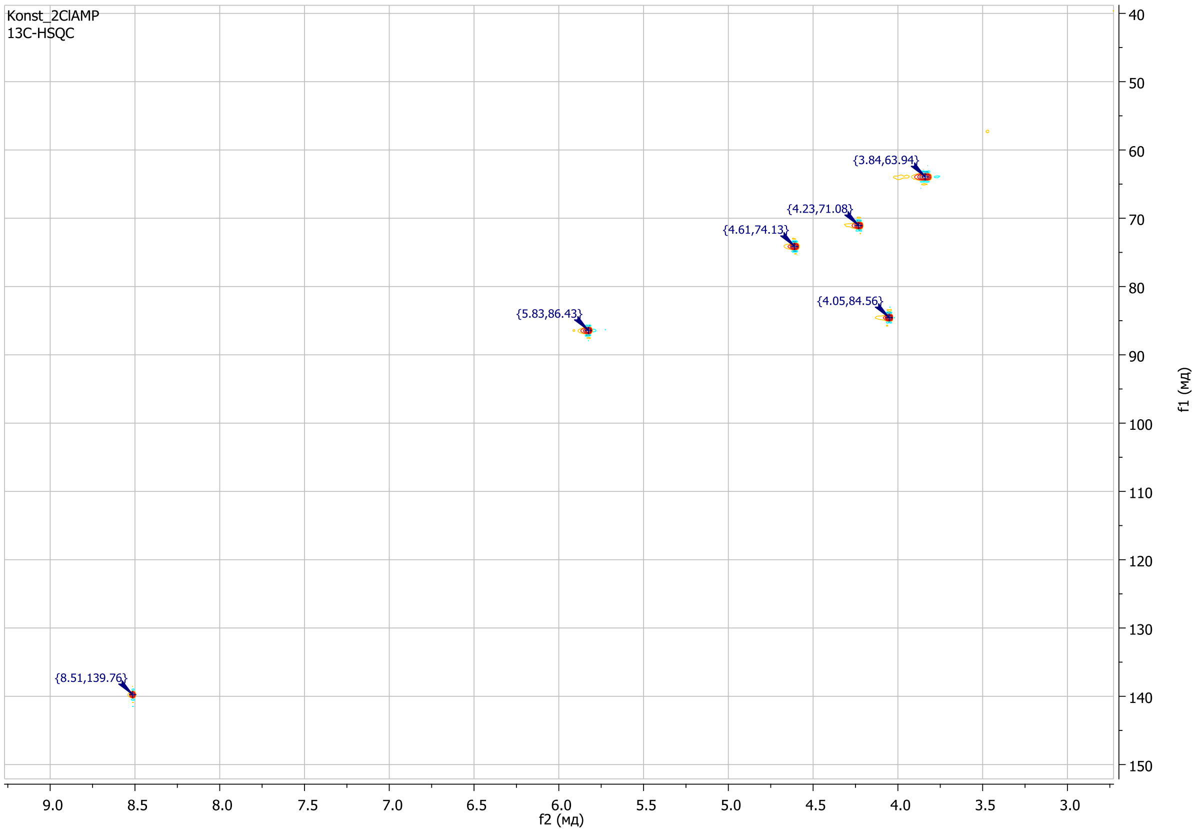

Figure S11. ^{1}H,^{13}C-HSQC spectrum of 2-Cl-AMP

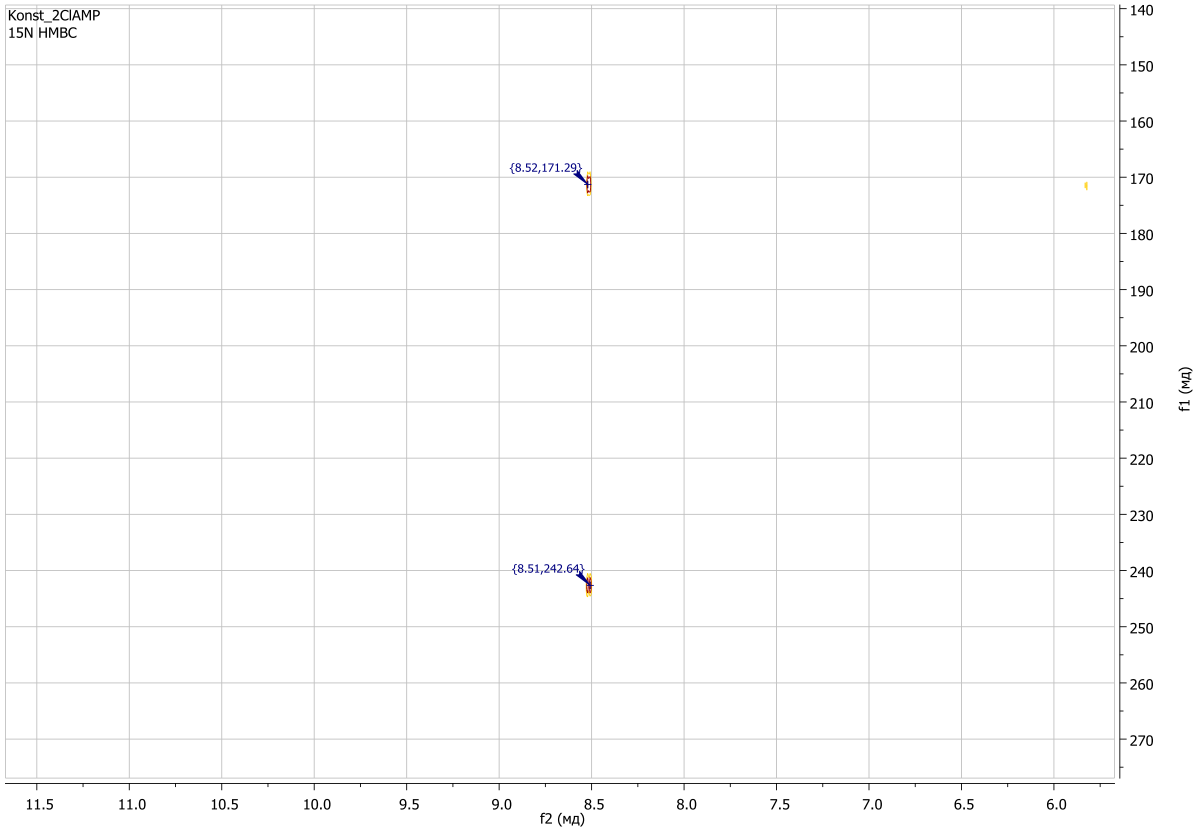

Figure S12. $^{1}H,^{15}N$-HMBC spectrum of 2-Cl-AMP

4. NMR data of Allop-MP

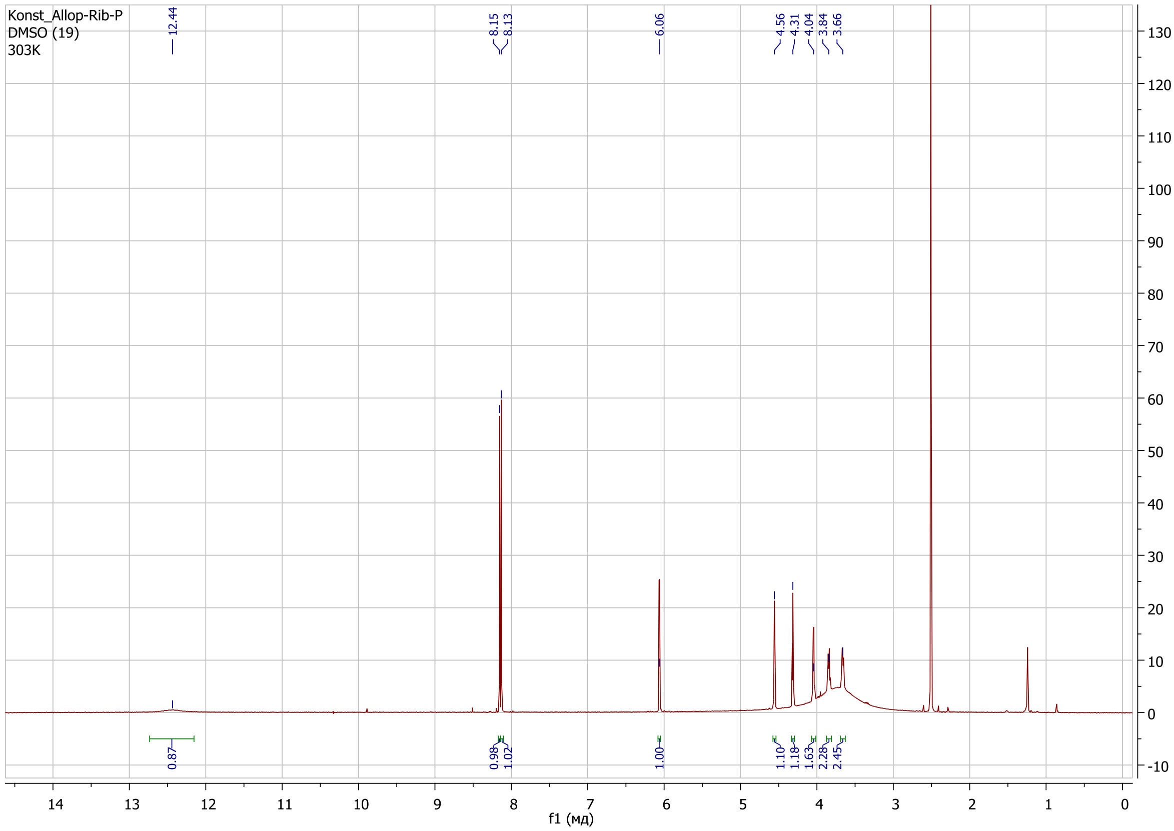

Figure S13. ^{1}H NMR spectrum of Allop-MP

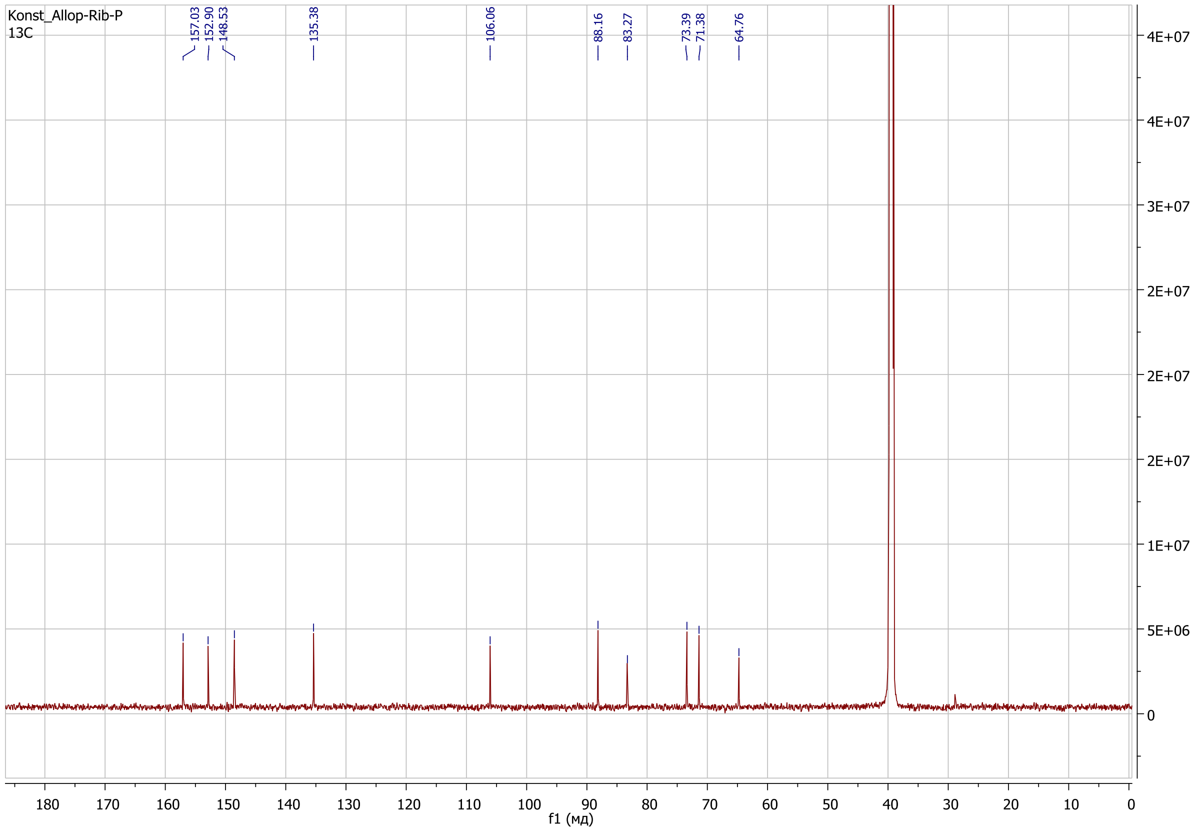

Figure S14. ^{13}C NMR spectrum of Allop-MP

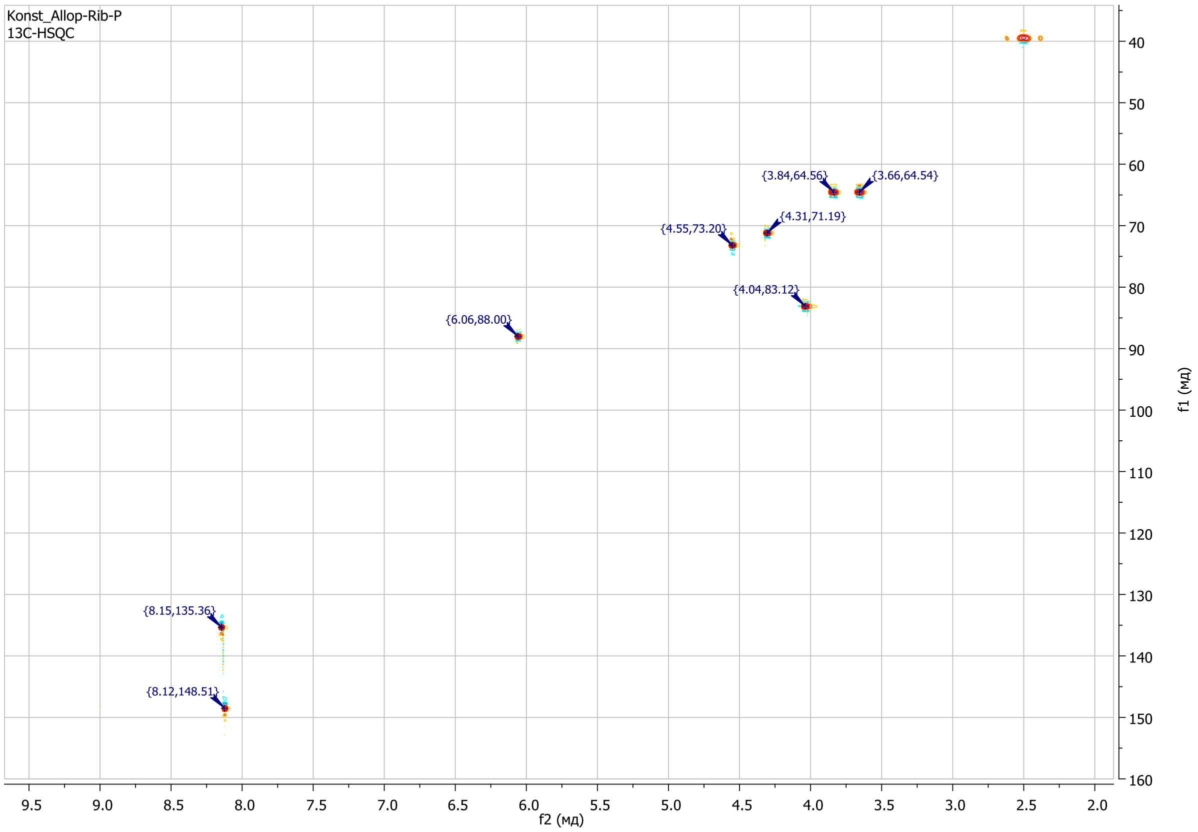

Figure S15. ^{1}H,^{13}C-HSQC spectrum of Allop-MP

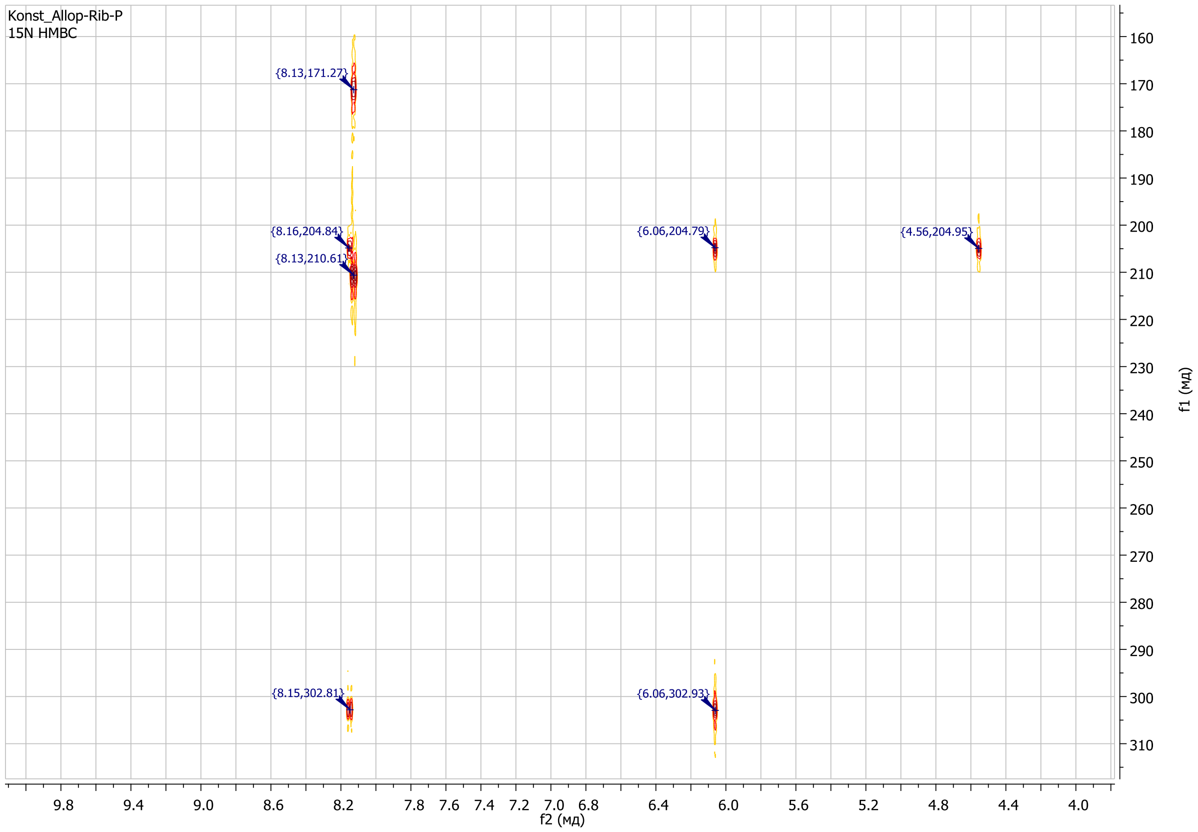

Figure S16. $^{1}H,^{15}N$-HMBC spectrum of Allop-MP

5. HPLC examples for nucleosides formation.

Waters 1525, column Ascentis Express C18, 2.7 µm, 3.0 x 75 mm, eluent A 0.1 % aqueous TFA, eluent B 0.1 % TFA / 70 % acetonitrile in water, detection at 254 nm, Waters 2489

Method 1: linear gradient elution from 0 to 10 % eluent B in eluent A, 20 min, flow rate 0.5 mL/min.

Method 2: elution 3 % eluent B in eluent A, flow rate 0.3 mL/min.

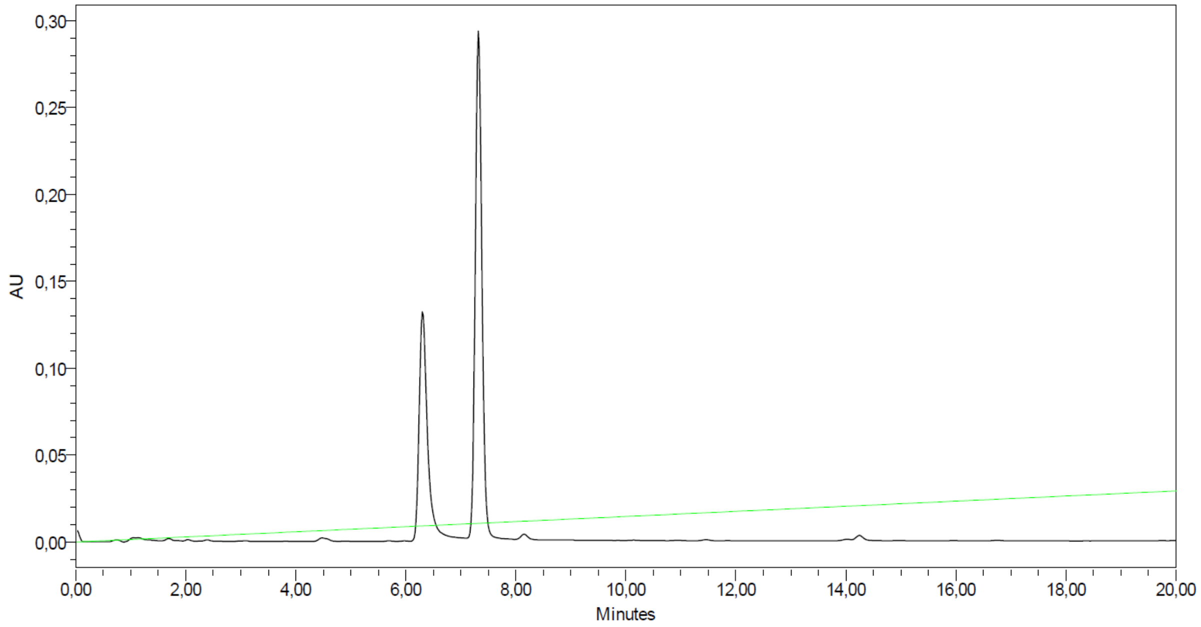

Figure S17. HPLC results for conversion 2-chloroadenine (RT = 6.3 min) to 2-Cl-AMP (RT = 7.4 min) at the presence of *Tth*APRT Method 1.

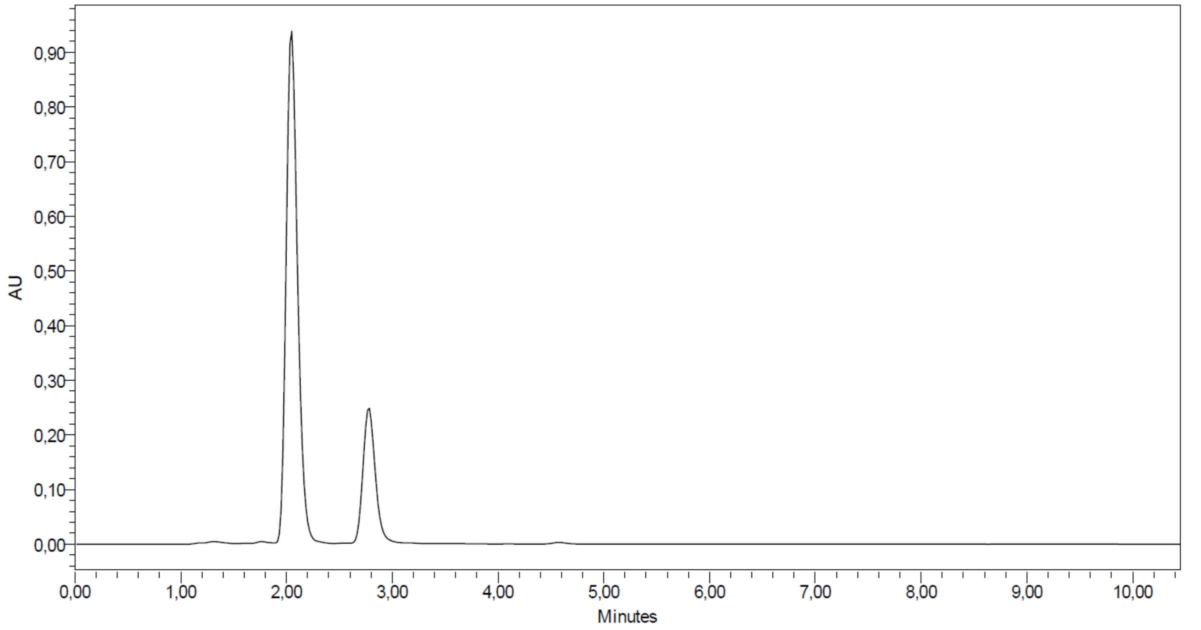

Figure S18. HPLC results for conversion 8-azaguanine (RT = 2.8 min) to 5-amino-3-(β-D-ribofuranosyl)-1,2,3-triazolo[4,5-d]pyrimidin-7-one 5'-monophosphate (RT = 2.0 min) at the presence of *Tth*HPRT Method 2.

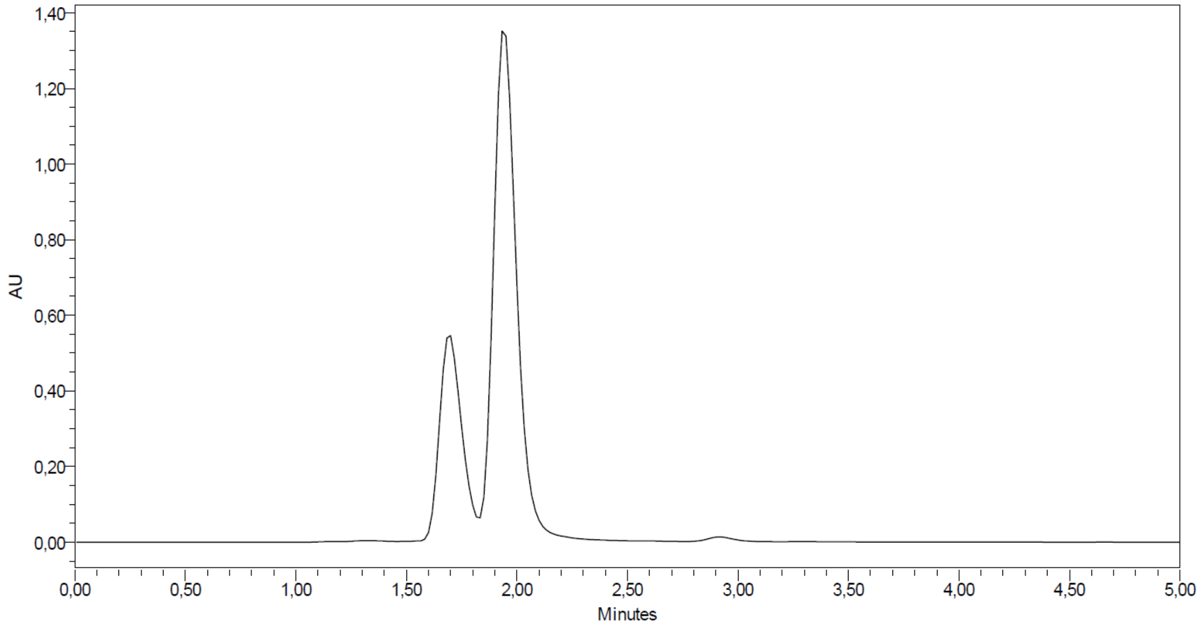

Figure S19. HPLC results for conversion adenine (RT = 2.0 min) to adenosine 5'-monophosphate (RT = 1.7 min) at the presence of *Tth*APRT Method 2.

6. Nucleotide sequence and corresponding amino acid sequence of the gene encoding the *Tth*HGPRT

ATGAAGGGCATGTTCACGCCGGGCAACGGACCCGTGCAGATCAGCGCCGAGG
CCATAAAGAAGCGGGTGGAGGAGCTGGGG**GGG**GAGATCGCCCGGGACTACCA
GGGCAAGACCCCTCACCTGATCTGCGTCCTGAACGGGGCCTTTATCTTCATGG
CCGACCTGGTGCGGGCCATCCCCCTGCCCCTCACCATGGACTTCATCGCCATC
AGCTCCTACGGGAACGCCTTCAAGTCCAGCGGGGAAGTGGAGCTTTTGAAGGA
CCTCCGCCTTCCCATCCACGGCCGGGACGTGATCGTGGTGGAGGACATCGTGG
ACACGGGGCTCACCCTCTCCTACCTTCTGGACTACCTCGAGGCCCGGAAGCCC
GCCTCCGTCCGCGTGGCCGCCCTCCTCTCCAAGCCCAGCCGCCGCCAGGTGG
AGGTGCCCATCCACTACCTGGGCTTTGAGATTGAGGACGCCTACGTCTACGGC
TACGGCCTGGACCGGGCCCAGTTTGACCGCAACCTGCCCTTCATCACCTCCAT
CCGCCCGGAGGAGGAATAA

MKGMFTPGNGPVQISAEAIKKRVEELG**G**EIARDYQGKTPHLICVLNGAFIFMADLVR
AIPLPLTMDFIAISSYGNAFKSSGEVELLKDLRLPIHGRDVIVVEDIVDTGLTLSYLLDY
LEARKPASVRVAALLSKPSRRQVEVPIHYLGFEIEDAYVYGYGLDRAQFDRNLPFITS
IRPEEEAAALEHHHHHH

The mutation (codone AGG → GGG) corresponding to amino-acid substitution Arg27Gly is boldfaced

Nucleotide sequence and corresponding amino acid sequence of the gene encoding the *Tth*APRT

ATGGTGAGGACCTACCCCGTTGAGATCGCCGGCGTGCGCAGGGAGCTCCCCAT
CGTCCAAGTGGGGCCGGGCGTGGCCGTGGCCCTGCTCAACCTCTTGGGGGAC
ACCGAGCTCACCGAGGCGGCCGCCGAGGCGCTGGCCAAGCGGCTTCCCCCGG
AGGTGGAGGTCCTGGTCACCCCCGAGGTCAAGGCCGTGCCCCTGGCCCACGC
CCTCTCCCGCATCACGGGCAAGCCCTACGTGGTGGCCCGCAAGACGGAGAAG
CCCTACATGATCAACCCCGTGAGCCGCCAGGTGCTCTCCATCACCACGGGGAA
GCCCCAGCTTCTGGTGCTGGACGGGGCCGACATCCCCCGGGTTCGGGGCAAG
AAGGTGGCCATCGTGGACGACGTGGTGTCCACCGGCTCCACCCTGGCGGGGC
TGAGGGAGCTCATTGAGAGCGTGGGGGGTGAGGTGGTCGCCGTCCTCGCCGT
CTTCACCGAGGGCACGCCCCGCCAGGACGTCGTGGCCCTCGGCCACCTCCCC
CTCTTCAAGCCGGAGTAGTAA

MVRTYPVEIAGVRRELPIVQVGPGVAVALLNLLGDTELTEAAAEALAKRLPPEVEVLV
TPEVKAVPLAHALSRITGKPYVVARKTEKPYMINPVSRQVLSITTGKPQLLVLDGADI
PRVRGKKVAIVDDVVSTGSTLAGLRELIESVGGEVVAVLAVFTEGTPRQDVVALGHL
PLFKPE

7. SDS-PAGE examples

Purification of the *Tth*HGPRT (SDS-PAGE documentation)

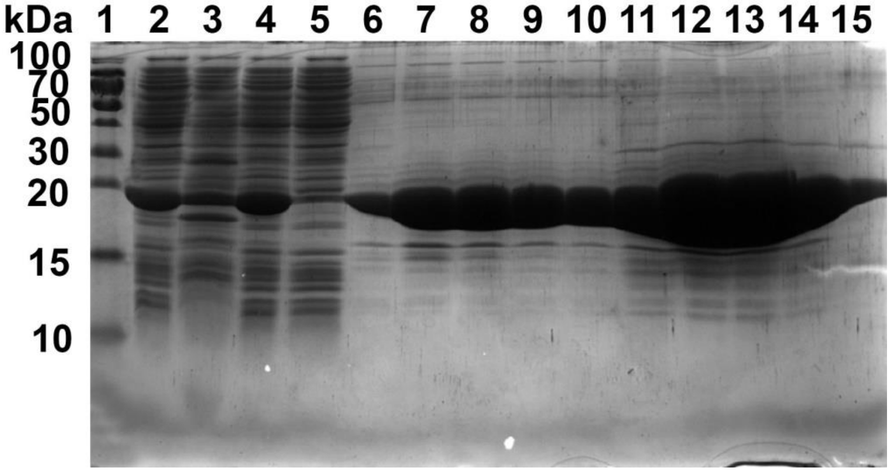

Figure S20. Protein fractions during immobilized metal affinity chromatography on Ni-IDA Sepharose column. 15% -SDS-PAGE. lane 1. molecular mass standards «PageRuler Broad Range Unstained Protein Ladder» (# 26630); lane 2, clarified cell supernatant; lane 3, denatured proteins after heating of clarified cell supernatant at 65°C for 10 min; lane 4, clarified cell supernatant after heating at 65°C for 10 min; lane 5, flow-through fraction; lanes 6 – 15, fractions after chromatography.

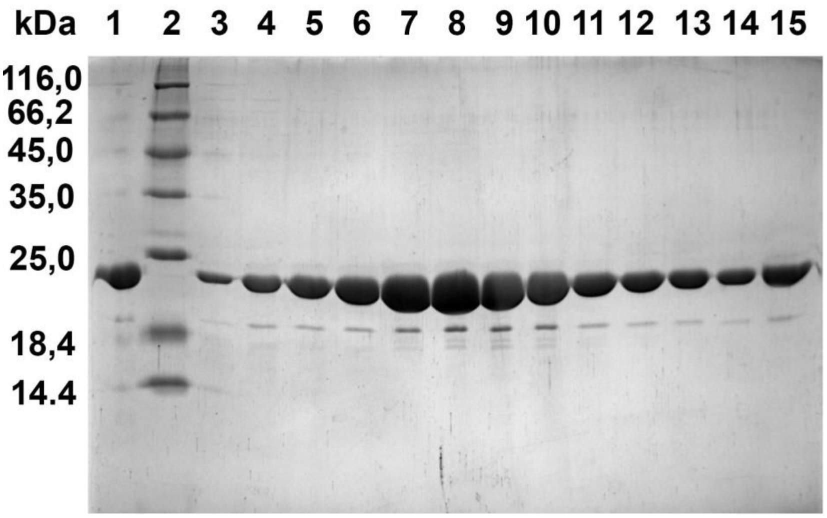

Figure 21. Protein fractions during size-exclusion chromatography on HiLoad 16/60 Superdex 75pg column. 15% -SDS-PAGE. lane 1, the combined fractions after immobilized metal affinity chromatography; lane 2, molecular mass standards «PageRuler Broad Range Unstained Protein Ladder» (# 26610); lane 3 – 15, fractions after chromatography.

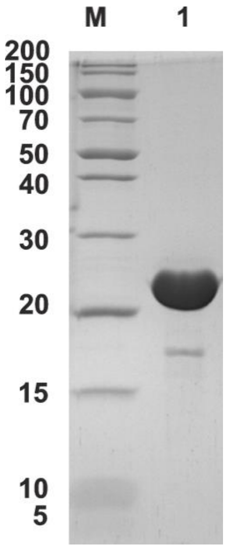

Figure S22. Purified *Tth*HGPRT. 15% -SDS-PAGE. lane M, molecular mass standards «PageRuler Broad Range Unstained Protein Ladder» (# 26630); lane 1, purified *Tth*HGPRT, 5 mkg

Purification of the *Tth*APRT (SDS-PAGE documentation)

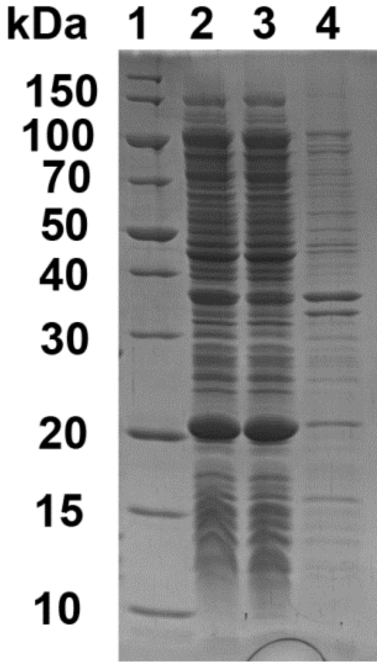

Figure S23. Protein fractions during hydrophobic chromatography on Phenyl Sepharose HP column. 15% -SDS-PAGE. . lane 1. molecular mass standards «PageRuler Broad Range Unstained Protein Ladder» (# 26630); lane 2, total cell lysate of the *E. coli* cells; lane 3, clarified cell supernatant; lane 4, the *E. coli* cells debris.

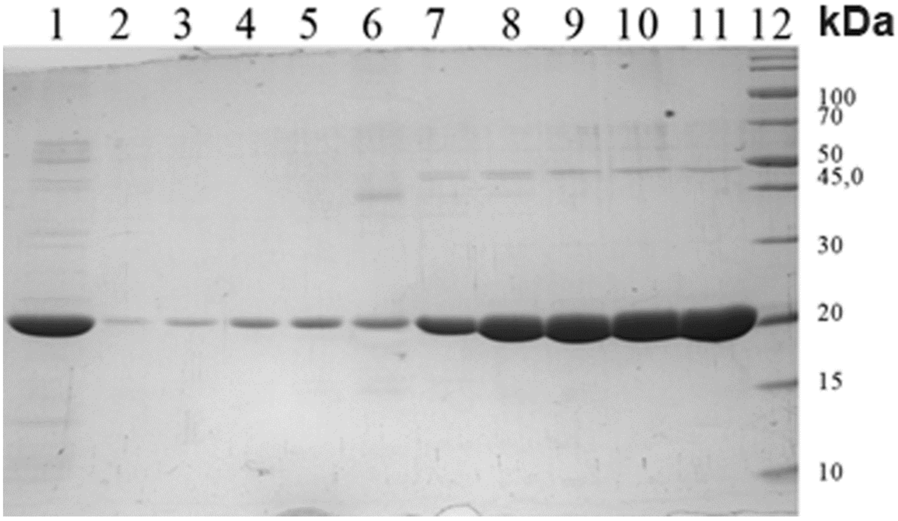

Figure S24. Protein fractions during hydrophobic chromatography on Phenyl Sepharose HP column. 15% -SDS-PAGE. lane 1, clarified cell supernatant after heating at 65°C for 10 min; lane 2 – 11, fractions after chromatography; lane 12 molecular mass standards «PageRuler Broad Range Unstained Protein Ladder» (# 26630).

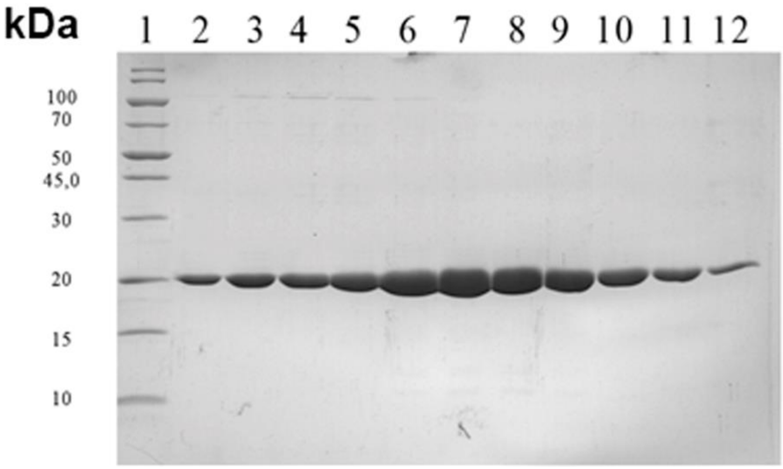

Figure S25. Protein fractions during size-exclusion chromatography on HiLoad 16/60 Superdex 200 pg column. 15% -SDS-PAGE. lane 1, molecular mass standards «PageRuler Broad Range Unstained Protein Ladder» (# 26630); lane 2 – 12, fractions after chromatography.

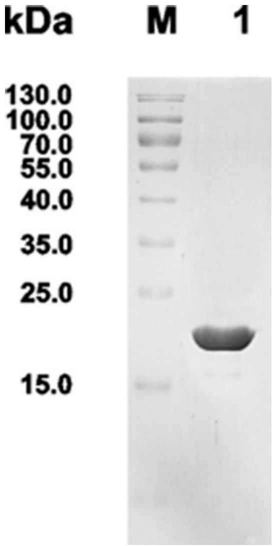

Figure S26. Purified *Tth*APRT. 15% -SDS-PAGE. lane 1, molecular mass standards «PageRuler Broad Range Unstained Protein Ladder» (# 26616). lane 2, purified *Tth*APRT, 5 mkg
